# Renewable energy infrastructure impacts biodiversity beyond the area it occupies

**DOI:** 10.1073/pnas.2208815119

**Published:** 2022-11-21

**Authors:** Bernardo B. Niebuhr, Daniela Sant’Ana, Manuela Panzacchi, Bram van Moorter, Per Sandström, Ronaldo G. Morato, Anna Skarin

**Affiliations:** ^a^Norwegian Institute for Nature Research, 7485 Trondheim, Norway; ^b^National Research Center for Carnivore Conservation, Chico Mendes Institute for the Conservation of Biodiversity, 12952-011 Atibaia, Brazil; ^c^Swedish University of Agricultural Sciences, 750 07 Uppsala, Sweden; ^d^Swedish University of Agricultural Sciences,901 83 Umeå, Sweden

Dunnett et al. (1) conclude that current and predicted priority areas for wind and solar energy development (renewable energy areas, REA) *overlap minimally* with important conservation areas (PA) in some regions of the globe. Their analysis assumes that land is a limiting resource to be shared among sustainability goals and focuses on area overlap between goals to assess their potential conflicts. However, conflicts may arise from impacts of infrastructure beyond their spatial delimitation. We add to their study (1) by highlighting that measures of area overlap are insufficient to encompass how complex social–ecological systems are affected by industrial activity.

The area infrastructure occupies does not say everything about its impacts—which may be indirect, accumulate, and affect larger areas. Here, we distinguish direct effects of infrastructure (habitat removal, as considered by Dunnett et al. (1)) from impacts, understood as functional responses of species, ecosystems, or human communities (2). In many ecosystems, the zone of influence (ZoI) of infrastructure extrapolates the occupied area and extends over several kilometers (3, 4). For example, roads modify small areas, yet they might produce animal avoidance over large distances (5) and disrupt gene flow among populations (6). Dunnett et al. calculated overlap ratios (OR) between priority PA and REA and indicated that the overlaps are underrepresented (<1) if weighted by the land available. Using their maps of priority areas, we calculated “impact OR” considering the proportion of priority PA that overlaps with assumed moderate and large, impacted zones, using buffers of 1 and 5 km around the priority REA. This results in larger OR for the ZoI, with impact OR overrepresented (>1) for most regions (Fig. 1). Therefore, although area overlap is small in some regions, infrastructure impact might be widespread (Fig. 2). Furthermore, multiple infrastructures usually cause cumulative impacts, which can be stronger, extend over larger areas, and last longer than impacts of single infrastructure (2, 7). Thus, we also suggest considering potential cumulative impacts of multiple RE and other infrastructure.

**Fig. 1.**
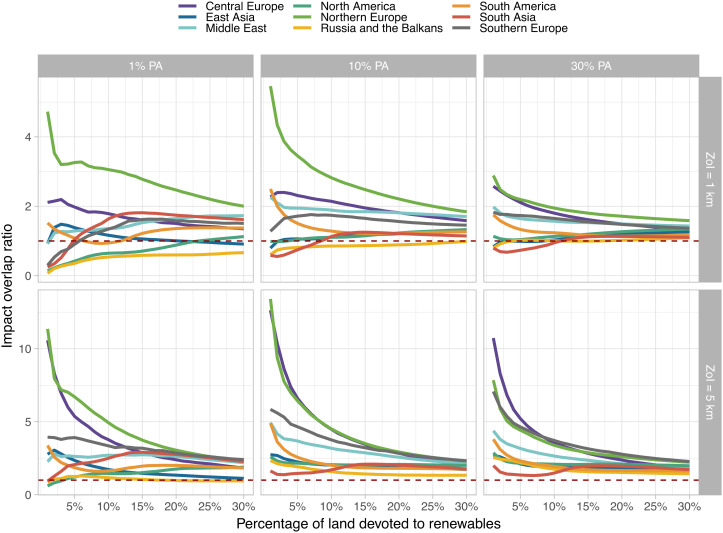
Overlap ratio between priority areas for biodiversity conservation (PA) and priority areas for wind energy development, considering a zone of influence (ZoI) of 1 and 5 km around priority renewable energy areas. Impact overlap ratios are higher than 1 (dashed line) for most regions and percentages of land devoted to renewables, which indicates the impact overlap is overrepresented (large), when weighted by the land available, contrary to the area overlap ratios presented in ref. 1. The results are similar for solar energy development. The extents of the ZoI considered here are just illustrative; they might vary for different species, systems, and processes and should be assessed together with the magnitude of the impacts.

**Fig. 2.**
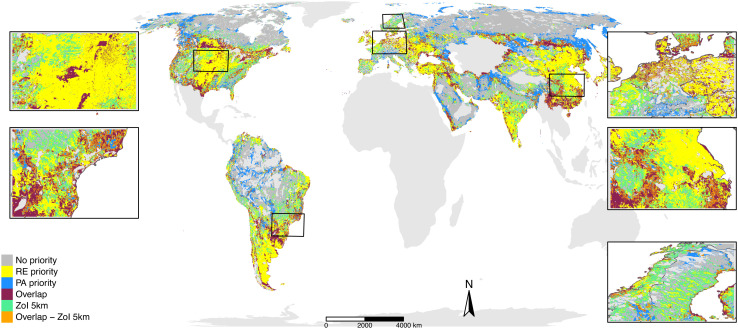
Overlap between the top 30% of land for wind energy priority expansion (renewable energy or RE priority) and the top 30% of land for protected area priority expansion (PA priority), including a ZoI of 5 km around the top 30% land for RE priority. The map is similar to Fig. 2*A* presented by Dunnet et al. (1) for wind power development; however, it also shows areas where the impact of RE priority sites might extrapolate the area they occupy (here a buffer of 5 km). The areas where this ZoI overlaps with PA priority areas (in orange) are much wider than only the areas of direct overlap (in red), and the ZoI of RE priority areas is also widespread outside PA priority areas, potentially impacting biodiversity outside important conservation areas. A new inset shows part of Scandinavia where the predicted area overlap is small but a series of conflicts between wind power development and Sami reindeer husbandry outside PA have been proliferating (8, 9).

Anthropogenic infrastructure also impacts ecosystems outside PA and indigenous and peasant livelihoods directly dependent on them. For instance, the area overlap predicted by Dunnett et al. (1) in Scandinavia is small (Fig. 2), yet the accelerated RE development has been affecting reindeer and Sami reindeer husbandry (3, 10), leading to environmental conflicts (8, 9). Similar impacts abound in the most biodiverse tropical areas (4, 9), which are partly absent from the analyses in ref. 1. In these cases, social–ecological impacts of RE are often considered minimal compared to their global benefits. Conflicts between RE and PA are not isolated and might have repercussions on other sustainable development goals aimed at increasing justice and reducing inequalities.

Therefore, impact assessments must include but not be limited to results such as presented in ref. 1. *Minimal overlap does not imply minimal impact*. Public policy should be designed with proper measures of impact, including areas indirectly impacted around infrastructure. Given the global pressure for rapid energy transition, we call for careful examination of impacts of renewable energy development on biodiversity and society, with wide interdisciplinary contribution and public participation.

## References

[1] S. Dunnett, R. A. Holland, G. Taylor, F. Eigenbrod, Predicted wind and solar energy expansion has minimal overlap with multiple conservation priorities across global regions. Proc. Natl. Acad. Sci. U.S.A. **119**, e2104764119 (2022). 35101973 10.1073/pnas.2104764119 PMC8832964

[2] M. P. Gillingham, G. R. Halseth, C. J. Johnson, M. W. Parkes, Eds., The Integration Imperative: Cumulative Environmental, Community and Health Effects of Multiple Natural Resource Developments(Springer International Publishing, 2016), 10.1007/978-3-319-22123-6.

[3] A. Skarin, B. Åhman, Do human activity and infrastructure disturb domesticated reindeer? The need for the reindeer’s perspective. Polar Biol. **37**, 1041–1054 (2014).

[4] P. Fan , Recently constructed hydropower dams were associated with reduced economic production, population, and greenness in nearby areas. Proc. Natl. Acad. Sci. U.S.A. **119**, e2108038119 (2022). 35131897 10.1073/pnas.2108038119 PMC8872755

[5] A. Torres, J. A. G. Jaeger, J. C. Alonso, Assessing large-scale wildlife responses to human infrastructure development. Proc. Natl. Acad. Sci. U.S.A. **113**, 8472–8477 (2016). 27402749 10.1073/pnas.1522488113 PMC4968732

[6] R. Holderegger, M. Di Giulio, The genetic effects of roads: A review of empirical evidence. Basic Appl. Ecol. **11**, 522–531 (2010).

[7] C. J. Johnson , Cumulative effects of human developments on Arctic wildlife. Wildl. Monogr. **160**, 1–36 (2005).

[8] D. Cambou, P. Sandström, A. Skarin, E. Borg, “Reindeer husbandry vs. wind energy” in Indigenous Peoples, Natural Resources and Governance (Routledge ed. 1, 2021), pp. 39–58.

[9] C. Lingaas, Wind farms in indigenous areas: The Fosen (Norway) and the Lake Turkana Wind Project (Kenya) cases (2021). Access in: http://opiniojuris.org/2021/12/15/wind-farms-in-indigenous-areas-the-fosen-norway-and-the-lake-turkana-wind-project-kenya-cases/.

[10] A. Skarin, P. Sandström, M. Alam, Out of sight of wind turbines-reindeer response to wind farms in operation. Ecol. Evol. **8**, 9906–9919 (2018). 30386585 10.1002/ece3.4476 PMC6202756

